# Surgery of the Genitourinary Organs

**Published:** 1908-03

**Authors:** 


**Surgery of the Genitourinary Organs.** By **J. W. S. Gouley,** M.D. Small 8 vo. pp. 531. New York: Rebman Co. (Price, $3.00).

There is little that is new in this book and its interest lies solely in its value as a historic work presenting the procedures of bygone years both in surgical and therapeutic treatments. Viewed in this light it is well worth the reading and is an excellent picture of what used to be and not what is. It would be not alone unfair, but absolutely criminal to criticise a book of this character, for it is in no sense intended as a textbook. As an exposition of the historic elements of genitourinary surgery it is excellent and might be read with benefit by students, provided they could so girt themselves about with self-control that they would be in no danger of becoming converts to some of the treatment suggestions, as for instance, leeching in epididymitis. There is much that is safe and a very great deal that is interesting in the book and it possesses the elegance of construction and the dignity for which Dr. Gouley's writings are noted.

